# The CT collateral map: collateral perfusion estimation and baseline lesion assessment after acute anterior circulation ischemic stroke

**DOI:** 10.1007/s11547-024-01941-5

**Published:** 2024-12-11

**Authors:** Hee Jong Ki, Hong Gee Roh, Jin Tae Kwak, In Seong Kim, Jeong Jin Park, Yoo Sung Jeon, Hyun Yang, Sumin Jung, Ji Sung Lee, Hyun Jeong Kim

**Affiliations:** 1 Department of Neurosurgery, Daejeon St. Mary’s Hospital, College of Medicine, The Catholic University of Korea, Seoul, Republic of Korea; 2 Department of Radiology, Konkuk University Medical Center, Konkuk University School of Medicine, Seoul, Republic of Korea; 3 DeepClue Inc., Daejeon, Republic of Korea; 4 School of Electrical Engineering, Korea University, Seoul, Republic of Korea; 5 Siemens Healthineers Ltd, Seoul, Republic of Korea; 6 Department of Neurology, Konkuk University Medical Center, Konkuk University School of Medicine, Seoul, Republic of Korea; 7 Department of Neurosurgery, Konkuk University Medical Center, Konkuk University School of Medicine, Seoul, Republic of Korea; 8 Clinical Research Center, Asan Institute for Life Science, Asan Medical Center, University of Ulsan College of Medicine, Seoul, Republic of Korea; 9 Department of Radiology, Daejeon St. Mary’s Hospital, College of Medicine, The Catholic University of Korea, 64 Daeheung-Ro, Jung-Gu, Daejeon Seoul, 34943 Republic of Korea

**Keywords:** Cerebrovascular disorders, Stroke, Ischemia, Collateral circulation, Diagnostic imaging, Perfusion imaging

## Abstract

**Purpose:**

To investigate the clinical feasibility of a CT collateral map compared with an MRA collateral map, focusing on collateral perfusion (CP) estimation and baseline lesion assessment in acute ischemic stroke (AIS).

**Materials and Methods:**

This retrospective analysis used selected data from a prospectively collected database. We generated CT collateral maps derived from CT perfusion, encompassing images of arterial, capillary, early venous (CMEV), late venous, and delay phases. Three raters assessed CP scores from MRA and CT collateral maps and CMEV lesion volumes. Lesion volumes of baseline diffusion-weighted imaging (bDWI) and cerebral blood flow rate (CBF) < 30% were automatically measured by the software. The agreement between MRA and CT collateral maps in CP estimation and the correlation between lesion volumes with a CBF < 30% and the CMEV for bDWI lesion volumes were analyzed.

**Results:**

One-hundred ten patients (mean age ± standard deviation, 71 ± 14; 60 women) with AIS due to steno-occlusion of the internal carotid and/or middle cerebral arteries were included. The agreement between the MRA and CT collateral maps in CP grading was excellent (weighted κ = 0.93; 95% CI, 0.90–0.97). The concordance correlation coefficients (CCCs) of the CBF < 30% and CMEV for bDWI lesion volumes were 0.76 (95% CI, 0.60–0.91) and 0.97 (0.95–0.98), respectively.

**Conclusion:**

The clinical feasibility of the CT collateral map is demonstrated by its significant correlation with the MRA collateral map in CP estimation and baseline lesion assessment.

## Introduction

In acute ischemic stroke (AIS) caused by large vessel occlusion, tissue and functional outcomes are primarily influenced by the collateral circulation [1, 2]. Prior research on recanalization therapies have demonstrated that robust collateral circulation is linked to reduced infarct expansion and improved functional recovery. Conversely, inadequate collateral circulation is associated with a higher risk of hemorrhagic complications and poor functional outcomes, frequently leading to futile treatments [3–9]. Thus, evaluating the collateral status is crucial for making informed treatment decisions in patients with AIS.

A novel multiphase collateral imaging derived from dynamic contrast-enhanced magnetic resonance angiography (DCE-MRA) and referred to as the MRA collateral map has recently been developed, along with a corresponding collateral perfusion (CP) grading system [10]. The MRA collateral map comprises five-phase images customized to the patient’s specific hemodynamics, offering insights into brain parenchymal perfusion and the status of collateral vessels. Clinical studies of the MRA collateral map have demonstrated its ability to predict both functional and tissue outcomes in patients with acute anterior circulation ischemic stroke [11, 12]. Yi et al. found that lesions identified by baseline diffusion-weighted imaging (DWI) with good CP status on the MRA collateral map did not expand. In contrast, lesions with intermediate and poor CP status were independently associated with lesion growth. When predicting final infarct size, the collateral ratio-calculated as the volume of the hypoperfused lesion in the capillary phase of the collateral map (CMC lesion volume) relative to the volume of the hypoperfused lesion in the early venous phase (CMEV lesion volume)-outperformed the conventional mismatch ratio, which compares the perfusion lesion volume with a threshold of > 6 s of the contralateral mean time-to-maximum (Tmax > 6 s lesion volume) to the baseline DWI (bDWI) lesion volume [12]. These results indicate that the MRA collateral map can offer precise information regarding baseline lesion, penumbra, and CP status in AIS patients caused by large vessel occlusion.

In AIS, CT-based imaging is commonly favored over MR-based imaging due to its speed and wide availability. However, CT-based imaging work-up for assessing baseline lesions and penumbras have certain limitations [13–17]. To address this, we adapted the principles used in generating the MRA collateral map to develop a CT collateral map derived from CT perfusion imaging. This study aimed to investigate the clinical feasibility of the CT collateral map by comparison with the MRA collateral map, specifically focusing on CP estimation and baseline lesion assessment.

## Materials and methods

### Study participants

**Fig. 1:**
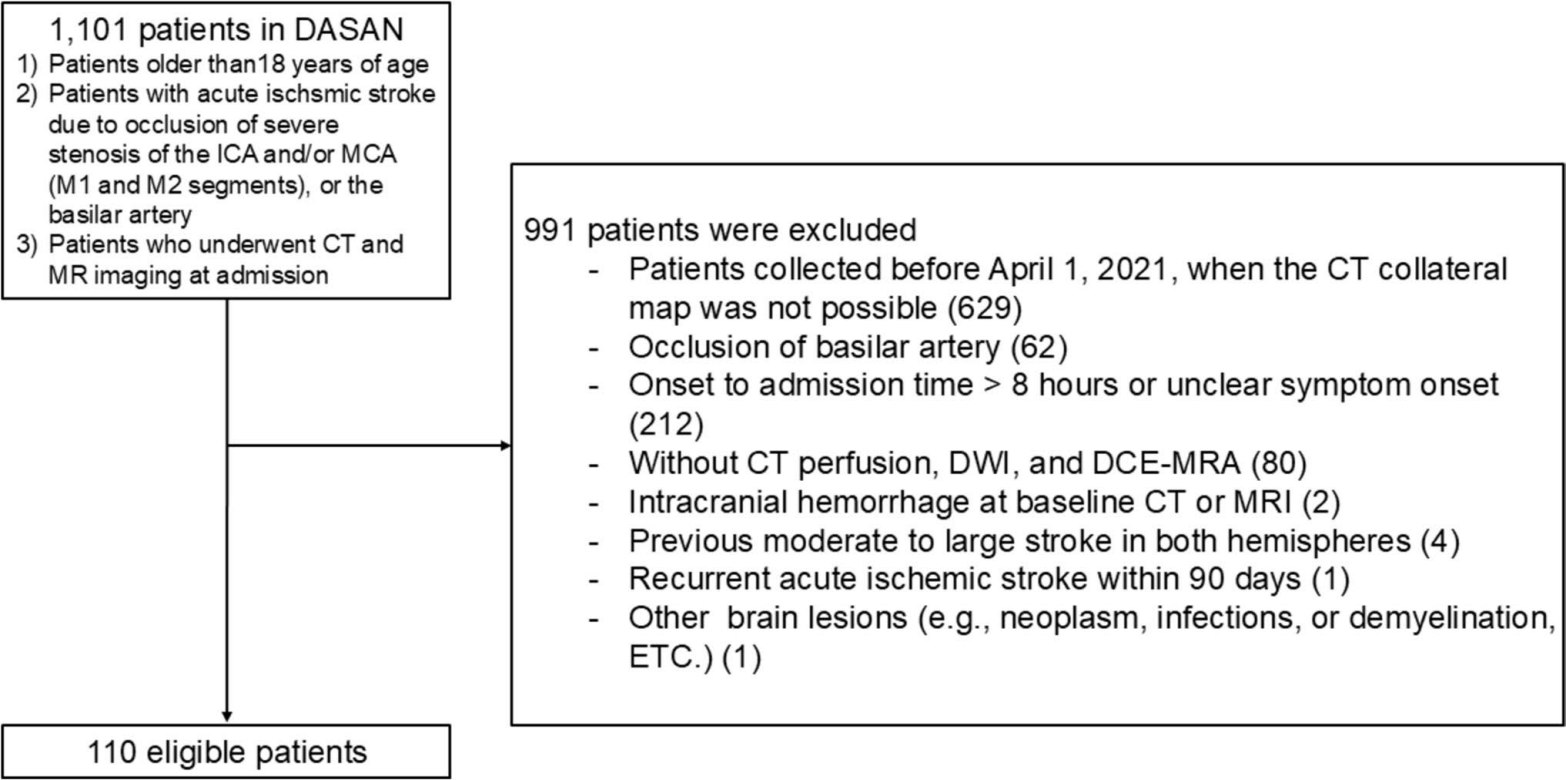
Flowcharts showing patient inclusion. DASAN = database of acute ischemic stroke analysis network, DCE-MRA = dynamic contrast-enhanced magnetic resonance angiography, DWI = diffusion-weighted imaging, ICA = internal carotid artery, MCA = middle cerebral artery

For this retrospective analysis, we selected patients from the ongoing Database of Acute ischemic Stroke Analysis Network (DASAN), prospectively gathered from two university hospitals starting January 1, 2016. The following DASAN inclusion criteria were used: (1) aged 18 years and older; (2) had AIS on DWI due to occlusion or severe stenosis of the internal carotid artery and/or the M1 or M2 segment of the middle cerebral artery (MCA) or the basilar artery; and (3) underwent brain CT imaging followed by MR imaging including DWI and DCE-MRA consequently at admission. Patients with steno-occlusion of the internal carotid artery and/or M1 or M2 segment of the MCA who were evaluated within 8 h of symptom onset and underwent CT perfusion, DWI, and DCE-MRA at admission were selected for this study. The exclusion criteria were as follows: patients whose data were collected before April 1, 2021, those for whom a CT collateral map was not possible, those with unclear symptom onset time, those with intracranial hemorrhage at baseline CT or MRI, those with a previous moderate to large stroke in both hemispheres, those who experienced recurrent AIS within 90 days, or those with other brain lesions (e.g., neoplasm, infection, or demyelination) (Fig. 1). Among the included patients, those who underwent follow-up DWI (f/uDWI) and angiography within 7 days and had unchanged stenoocclusive arterial lesions on follow-up angiography were selected for subgroup analysis to compare the predictive performance for f/uDWI lesion volume between the CMC lesion volume and the Tmax > 6 s lesion volume. Patient evaluations included demographic data, medical history, vascular risk factors, routine blood tests, brain imaging, and cardiological tests. Stroke severity was assessed using the National Institutes of Health Stroke Scale (NIHSS).

### Imaging protocol and postprocessing for generating of the CT and MRA collateral maps

**Fig. 2:**
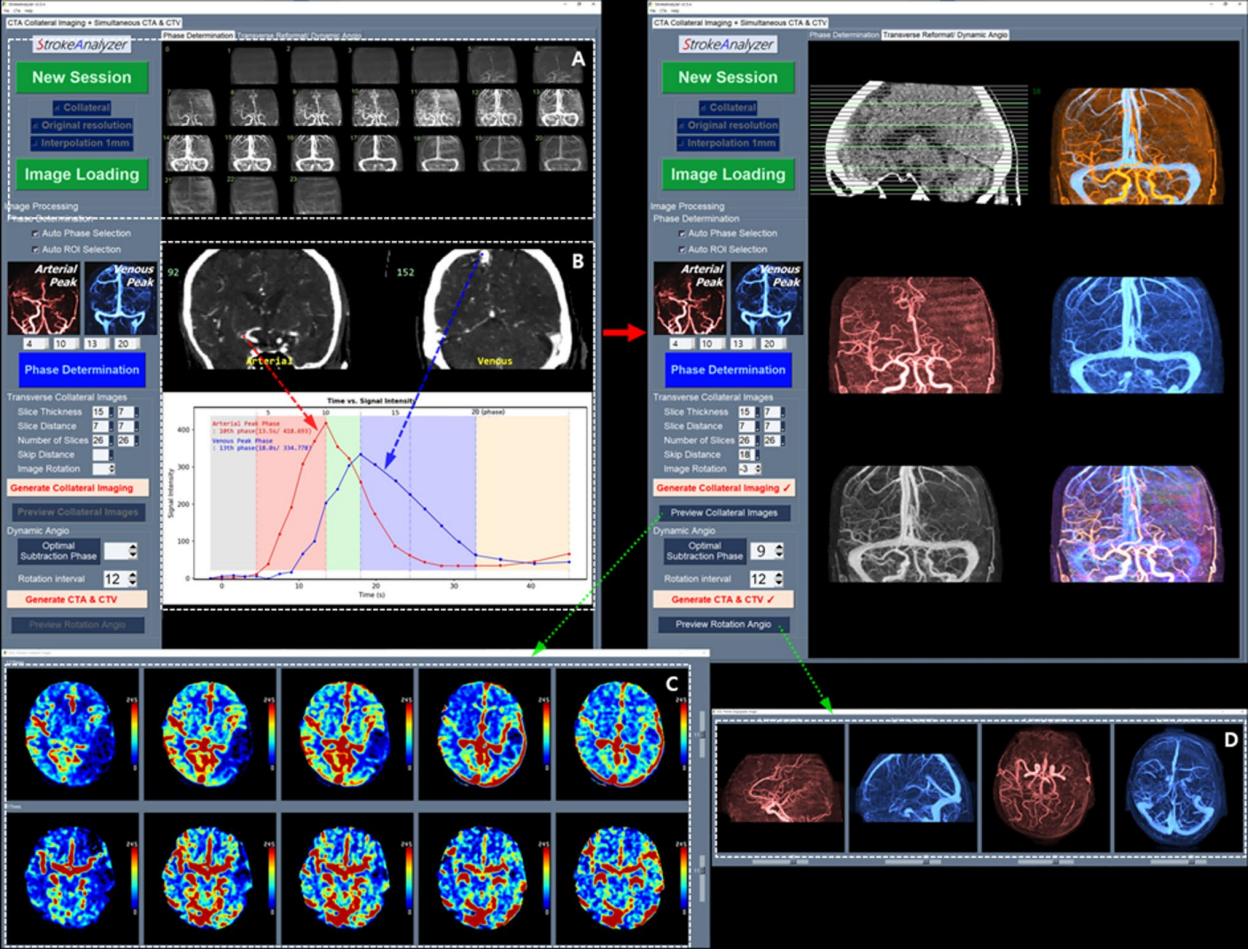
Postprocessing sequences for the CT collateral map using CT perfusion source images: (1) opening and reading DICOM source images of CT perfusion; (2) storing all acquired CT perfusion image data in a 4-dimensional matrix; (3) creating maximum intensity projection images for each time point representing bolus passage for each phase (**A**); (4) obtaining arterial and venous time-intensity curves for regions of interest on the major arteries (the middle cerebral artery, anterior cerebral artery or terminal internal carotid artery) in the nonischemic hemisphere and the superior sagittal sinus by plotting intensity changes in the region of interest (**B**); (5) separating the 4-dimensional images from CT perfusion into 5-phase data sets based on bolus passage status, plotted on the time-intensity curves (arterial phase = from the beginning of arrival of the contrast in the major artery to the arterial bolus peak; capillary phase = from just past the arterial bolus peak to just before the venous bolus peak in the superior sagittal sinus; early venous phase = first half of the venous phase from the venous bolus peak to the starting point of the venous plateau; late venous phase = second half of the venous phase; delay phase = from just past the venous phase to the end of the dynamic phase); and (6) reformatting the image data sets of each 5 phases into 5-phase axial image sets (CT collateral map) by averaging the intensities with the desired reconstruction parameters (slice range covering nearly the entire brain, slice thickness ranging from 7 to 15 mm, slice distance, and number of slices) (**C**). Using the optimal signal difference phase (the time point at which the maximum signal difference between the arterial and venous signal intensity-time curves in the arterial phase), color-coded arteriogram, venogram and dynamic angiography were simultaneously generated (**D**). The postprocessing time from loading source images of CT perfusion to generating the CT collateral map and angiographies was approximately 5 min

Noncontrast brain CT, head and neck CT angiography, and CT perfusion were conducted using dual-source CT scanners. The CT perfusion protocol involved 49.5-s (s) acquisition, consisting of 18 scans with a 1.5-s interval, followed by 3 scans with a 3-s interval, 3 scans with a 4.5-s interval at 70 kVp and 150 mAs, 96 × 2 × 0.6-mm detector collimation and 114 mm *z*-axis coverage. Images were acquired with a 7-s delay after injection of 45 ml of iodinated contrast medium with 350 mg iodine/ml at a rate of 5 mL/s, followed by a saline chase of 40 ml at a rate of 5 mL/s. We developed an in-house software developed using Python (version 3.8.10) to generate a CT collateral map, 3-dimensional color-coded arterio- and venographies, and dynamic angiography. The sequential postprocessing steps for the CT collateral map and angiographies are demonstrated in Fig. 2.

MRI was performed with 3-Tesla MRI scanners with acquisition parameters similar to those used in the previous study [10]. Utilizing source data from DCE-MRA, 3-dimensional rotational arteriography was conducted to determine the arterial status, and an MRA collateral map was reconstructed to evaluate the CP status. The MRA collateral map was generated using an in-house program following methods previously published [10], similar to those used for the CT collateral map.

### Collateral perfusion grading system and imaging analysis

Baseline and follow-up DWI lesion volumes were quantified using MEDIHUB STROKE software (version 2.1.0; JLK Inc., Seoul, Korea) [18]. The lesion growth ratio was calculated as the ratio of the f/uDWI lesion volume to the bDWI lesion volume, with lesion growth was defined as a lesion growth ratio ≥ 1.2 to account for the impact of vasogenic edema on the follow-up lesion volume. Given the perfusion thresholds of < 30% of the contralateral mean cerebral blood flow rate (CBF < 30%) and Tmax > 6 s are generally utilized for estimating the ischemic core and penumbra, respectively [19–22], the lesion volumes of baseline CBF < 30% (CBF < 30% lesion volume) and Tmax > 6 s were automatically computed using RAPID software. The mismatch ratio was defined as the ratio of Tmax > 6 s lesion volume to CBF < 30% lesion volume.

**Fig. 3:**
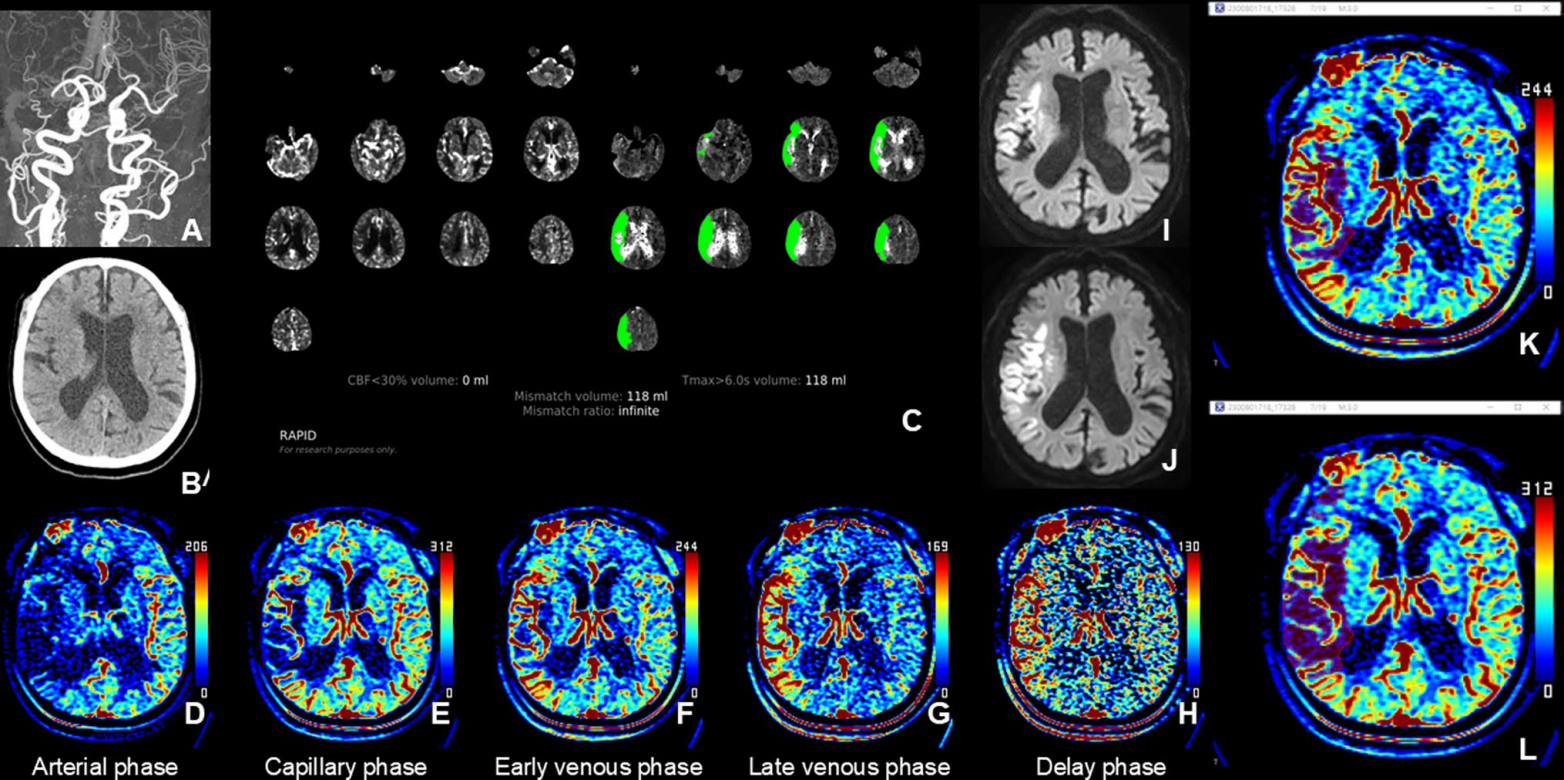
Images of an 82-year-old woman with occlusion of the right middle cerebral artery (MCA) demonstrated on CT angiography (**A**). This patient had a premorbid modified Rankin Scale (mRS) score of 0, and her National Institutes of Health Stroke Scale (NIHSS) score at admission was 12. She received intravenous thrombolysis followed by intraarterial thrombectomy, resulting in complete recanalization of the occluded artery. Her 90-day mRS score was 3. Upon admission, a brain CT scan (**B**) showed indistinct differentiation between the cortical gray matter and white matter in the right temporal and insular regions, making it difficult to precisely determine the extent of the baseline lesion. CT perfusion analysis using RAPID software (RAPID, RapidAI®, Menlo Park, CA, USA) (**C**) indicated a baseline lesion volume of 0 ml, as measured by a relative cerebral blood flow threshold of < 30%, meaning the baseline lesion could not be identified. The CT collateral map, generated from the CT perfusion images (**D**–**H**) at admission, indicated poor collateral perfusion (collateral perfusion score of 1: collateral perfusion delay involving more than half of the MCA territory in the early venous phase and less than half in the late venous phase). Diffusion-weighted imaging (DWI) (**I**), performed immediately after the CT scan, revealed acute infarct signals in the right MCA territory. The extent of the DWI lesion at admission closely corresponded with the hypoperfused area visible up to the early venous phase on the CT collateral map (**F**). This suggests that even without DWI, the baseline lesion can be identified using a CT collateral map. The baseline lesion shows slight evolution on DWI on day 1 after recanalization of the occluded MCA (**J**). **K** and **L** are images shown on Medical Image Processing, Analysis, and Visualization (MIPAV; version 7.1.1; National Institutes of Health). These images display the hypoperfused lesion highlighted in the early venous (**K**) and capillary (**L**) phases, respectively, for assessing the hypoperfused lesion volume on the collateral map

Three raters (H.G.R., with 23 years of experience as a neuroradiologist, H.J.K., with 8 years of experience as a neurosurgeon, and N.I.S., as fourth-year neurosurgery resident) who were blinded to all clinical and other imaging data independently graded the CP scores of both CT and MRA collateral maps in a blinded manner on two separate occasions, one week apart. The CP scores were as follows: 5, excellent; 4, good; 3, intermediate to good; 2, intermediate to poor; 1, poor; and 0, very poor (Table 1) [11, 12]. Final CP scores were determined by consensus among the raters. The CMC and CMEV lesion volumes were also measured by the three raters using Medical Image Processing, Analysis, and Visualization (MIPAV; version 7.1.1; National Institutes of Health, Bethesda, MD) as described in a previous study [12]. In the CMC, regions within the ischemic hemisphere with reduced perfusion compared to the contralateral hemisphere, and in the CMEV, regions of persisted hypoperfusion from the CMC were manually delineated on each slice using the freehand drawing brush tool (Fig. 3). The MIPAV software then automatically calculated the volume of the delineated areas across all slices by converting each outlined region into a voxel of interest. The final CMC and CMEV lesion volumes were determined by averaging the volumes measured by the three raters. The collateral ratio was calculated as the ratio of the CMC lesion volume to the CMEV lesion volume. In cases where the DWIs, CBF < 30%, and Tmax > 6 s lesion volumes measured by the software were 0 ml due to small lesions or when the collateral perfusion status was too favorable to detect hypoperfused lesions on the CT collateral map, volume values were considered 0.1 ml for statistical analysis.

**Table 1:** Collateral perfusion grading system for analysis of the CT and MRA collateral maps

Collateral perfusion score	Description of collateral perfusion status
5 (Excellent)	No or small^*^ collateral perfusion delay† in the ischemic MCA territory in the capillary phase regardless of the collateral perfusion status in the arterial phase
4 (Good)	Collateral perfusion delay ≤ ½ of the ischemic MCA territory in the capillary phase AND no or small collateral perfusion delay in the early venous phases
3 (Intermediate to good)	1. Collateral perfusion delay ≤ ½ of the ischemic MCA territory in the capillary and early venous phases 2. Collateral perfusion delay > ½ of the ischemic MCA territory in the capillary phase AND no or small collateral perfusion delay in the early venous phase
2 (Intermediate to poor)	Collateral perfusion delay > ½ of the ischemic MCA territory in the capillary phase AND ≤ ½ of the ischemic MCA territory in the early venous phase
1 (Poor)	Collateral perfusion delay > ½ of the ischemic MCA territory in the early venous phase AND ≤ ½ of the ischemic MCA territory in the late venous phase
0 (Very poor)	Collateral perfusion delay > ½ of the ischemic MCA territory in the late venous phase

MCA, middle cerebral artery

^*^^“^Small” refers an area less than one-eighth of the middle cerebral artery (MCA) territory, which is divided into regions: the insula, subcortical structures (including the basal ganglia and internal capsule), and the M1–M6 areas of the Alberta Stroke Programme Early CT Scores (ASPECTS). In cases where the collateral perfusion delay is visually ambiguous or borderline, particularly when it affects about half of the MCA territory, the collateral perfusion score is determined by counting the number of regions with observed delays. For instance, if the collateral perfusion delay impacts more than half of the MCA territory, it means that more than 4 out of the 8 regions within the MCA territory are involved

^†^In the capillary phase, the collateral perfusion delay in the ischemic MCA territory is assessed by comparing it to the perfusion in the contralateral MCA territory. In the early venous, late venous, and delay phases, any region that consistently shows a collateral perfusion delay, as noted in the capillary phase, is classified as having a collateral perfusion delay in the ischemic MCA territory

### Statistical analysis

Patient characteristics are presented as the mean ± standard deviation or median (interquartile range [IQR]) for continuous variables, or as the number of patients (%) for categorical variables. Differences in the distribution of patient characteristics across CP scores were analyzed using the Chi-square test, Fisher’s exact test, ANOVA and the Kruskal‒Wallis test, as appropriate. The interrater reliabilities for CP grading of both CT and MRA collateral maps and the measurement of hypoperfused lesion volumes in the CMC and CMEV were assessed using Kendall’s coefficient of concordance for an ordinal response and concordance correlation coefficient (CCC) for a continuous response, respectively. The agreement between the CP scores of the CT and MRA collateral maps was evaluated using the weighted Cohen’s kappa coefficient. To assess the accuracy of bDWI and f/uDWI lesion volumes between the conventional perfusion thresholds (CBF < 30% and Tmax > 6 s lesion volumes) and CT collateral map values (CMC and CMEV lesion volumes), CCC and linear regression analyses were performed. The correlation of the mismatch and collateral ratios with the lesion growth ratio was determined using the CCC. Significance levels were set at a 2-tailed *P* value less than 0.05. Statistical analyses were performed using SAS 9.4 (SAS Institute, Cary, NC).

## Results

### Participant characteristics and CP estimation

Among the 472 patients between April 1, 2021, and September 30, 2023, in DASAN, 110 were included in this study (Fig. 1). The mean age was 71 years ± 14 (range, 33–103 years), 55% were women (50 men and 60 women), the median baseline NIHSS score was 10 (IQR, 4–14), the median bDWI lesion volume was 12.6 ml (IQR, 2.9–34.8 ml), the median onset-to-door time was 70 min (IQR, 32–149 min), the median onset-to-CT time was 101 min (IQR, 47–175 min), the median onset-to-MRI time was 153 min (IQR, 98–235 min), and the median time interval from CT to MRI was 51.0 min (IQR, 41.0–66.0 min).

CT collateral maps were generated for all patients. MRA collateral maps were generated for 104 of the 110 patients, with 6 patients excluded due to loss of source data for DCE-MRA. The interrater correlation among the three raters showed almost perfect agreement for CP grading of the CT and MRA collateral maps (Kendall’s coefficient of concordance for ordinal response = 0.98; 95% CI: 0.97, 0.99 for both). The agreement between the CP scores of the CT and MRA collateral maps in 104 patients was almost perfect (weighted κ = 0.93; 95% CI: 0.90, 0.97). The baseline clinical characteristics for each CP grade of the CT collateral map are presented in Table 2. The absence of atrial fibrillation, lower baseline NIHSS scores, smaller baseline DWI lesion volumes, and favorable functional outcomes were associated with higher CP grades (all *P* < 0.001). Occlusion rather than stenosis and a higher number of occluded arteries were related to lower CP grades (*P* < 0.001).

**Table 2:** Patient characteristics (*n* = 110) according to the collateral perfusion grade based on the CT collateral map

Characteristics	Collateral perfusion grade based on the CT collateral map						*P* value
	CPS 0	CPS 1	CPS 2	CPS 3	CPS 4	CPS 5	
No. of patients	10	18	28	25	9	20	
Men, *n* (%)	4 (40.0)	7 (38.9)	14 (50.0)	12 (48.0)	5 (55.6)	8 (40.0)	0.937
Age (years)^*^	70 ± 11	70 ± 15	72 ± 15	72 ± 14	69 ± 17	71 ± 11	0.979
Glucose at admission (mg/dl)^†^	146 (108–172)	136 (109–159)	124 (102–147)	131 (115–168)	112 (105–153)	119 (108–144)	0.662
Systolic blood pressure at admission (mmHg)^*^	146.1 ± 19.5	148.1 ± 30.2	140.5 ± 22.4	153.1 ± 22.9	138.8 ± 13.7	157.8 ± 26.3	0.135
Diastolic blood pressure at admission (mmHg)^*^	75.0 ± 15.4	85.6 ± 16.4	80.2 ± 16.3	83.2 ± 17.4	88.7 ± 16.8	81.6 ± 18.4	0.503
Risk factors, *n* (%)							
Hypertension	7 (70.0)	14 (77.8)	15 (53.6)	16 (64.0)	7 (77.8)	12 (60.0)	0.571
Diabetes	3 (30.0)	9 (50.0)	7 (25.0)	5 (20.0)	1 (11.1)	5 (25.0)	0.296
Hyperlipidemia	2 (20.0)	1 (5.6)	8 (28.6)	3 (12.0)	0 (0.0)	5 (25.0)	0.199
Atrial fibrillation	7 (70.0)	11 (61.1)	18 (64.3)	8 (32.0)	1 (11.1)	1 (5.0)	< 0.001
Current smoker	1 (10.0)	1 (5.6)	4 (14.3)	7 (28.0)	2 (22.2)	2 (10.0)	0.402
Daily alcohol consumption	0 (0.0)	1 (5.6)	0 (0.0)	1 (4.0)	0 (0.0)	0 (0.0)	0.622
Previous TIA	0 (0.0)	0 (0.0)	0 (0.0)	0 (0.0)	0 (0.0)	0 (0.0)	
Previous stroke	1 (10.0)	2 (11.1)	6 (21.4)	3 (12.0)	1 (11.1)	1 (5.0)	0.736
Previous ischemic heart disease	2 (20.2)	3 (16.7)	3 (10.7)	2 (8.0)	0 (0.0)	3 (15.0)	0.757
Peripheral artery disease	0 (0.0)	0 (0.0)	0 (0.0)	0 (0.0)	0 (0.0)	0 (0.0)	
Baseline NIHSS score^†^	16 (13–19)	15 (12–19)	12 (9–15)	6 (4–11)	5 (3–5)	1 (0.2)	< 0.001
Baseline DWI lesion volume (ml)^†^	136 (100–149)	54 (23–81)	15 (10–25)	12 (4–21)	3 (1–5)	1 (0–1)	< 0.001
Site of steno-occlusion							< 0.001
ICA and/or MCA stenosis > 50%	0 (0.0)	0 (0.0)	2 (7.1)	2 (8.0)	5 (55.6)	17 (85.0)	
ICA occlusion	0 (0.0)	0 (0.0)	0 (0.0)	2 (8.0)	0 (0.0)	0 (0.0)	
M1 occlusion	2 (20.0)	7 (38.9)	11 (39.3)	4 (16.0)	3 (33.3)	1 (5.0)	
M2 occlusion	0 (0.0)	2 (11.1)	6 (21.4)	13 (52.0)	1 (11.1)	2 (10.0)	
ICA and M1 occlusion	6 (60.0)	6 (33.3)	5 (17.9)	4 (16.0)	0 (0.0)	0 (0.0)	
ICA and MCA occlusion	0 (0.0)	1 (5.6)	4 (14.3)	0 (0.0)	0 (0.0)	0 (0.0)	
MCA and ACA occlusion	0 (0.0)	1 (5.6)	0 (0.0)	0 (0.0)	0 (0.0)	0 (0.0)	
ACA or PCA occlusion combined with ICA and/or MCA occlusion	2 (20.0)	1 (5.6)	0 (0.0)	0 (0.0)	0 (0.0)	0 (0.0)	
Functional outcome^‡^							< 0.001
Favorable	0 (0.0)	6 (33.3)	12 (42.9)	11 (50.0)	6 (66.7)	18 (94.7)	
Unfavorable	10 (100.0)	12 (66.7)	16 (57.1)	11 (50.0)	3 (33.3)	1 (5.3)	

The mean overall patient age was 71 years ± 14 (standard deviation) years (50 men and 60 women). Unless otherwise noted, the data represent the number of patients, with percentages shown in parentheses

^*^Data represent the means ± standard deviations

^†^Data are presented as medians, with interquartile ranges shown in parentheses

^‡^Favorable functional outcome was defined as an modified Rankin scale (mRS) score of ≤ 2 and unfavorable functional outcome as an mRS score of > 2 in day 90

ACA, anterior cerebral artery; CPS, collateral perfusion score; DWI, diffusion-weighted imaging; ICA, internal carotid artery; M1, M1 segment of the middle cerebral artery; M2, M2 segment of the middle cerebral artery; NIHSS, National Institutes of Health Stroke Scale; TIA, transient ischemic attack

### Accessibility of the CT collateral map for baseline and final lesions

**Fig. 4:**
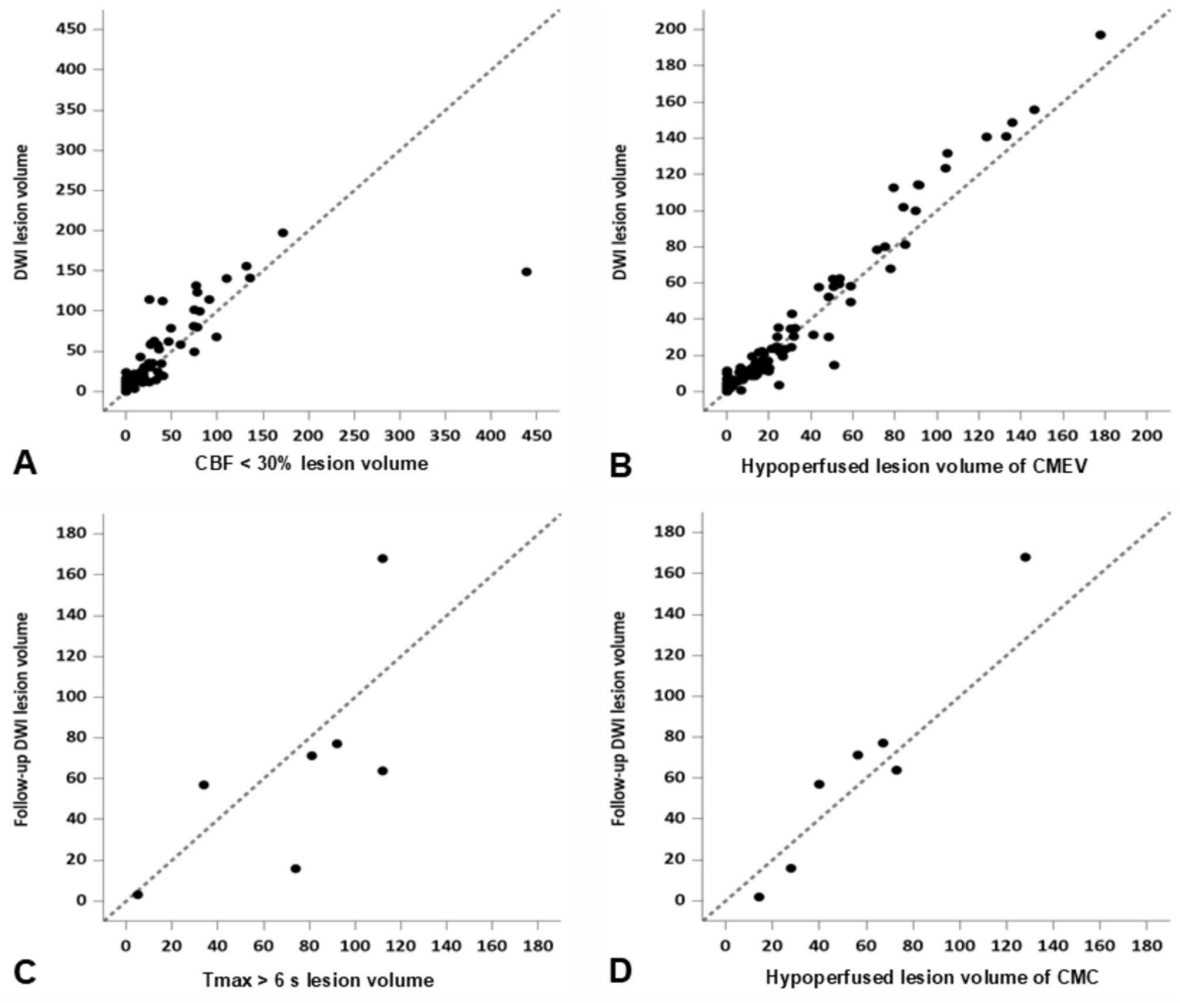
Scatterplots for the concordance correlation coefficient of the lesion volumes with a threshold of < 30% of the contralateral mean cerebral blood flow rate (CBF < 30%) and hypoperfused lesion volume in the early venous phases of collateral maps (CMEV) with the baseline diffusion-weighted imaging (DWI) lesion volumes (**A** and **B**) and the lesion volumes of > 6 s of the contralateral mean time-to-maximum (Tmax > 6 s) and hypoperfused lesion volume in the capillary phases of collateral maps (CMC) with the follow-up DWI lesion volumes (**C** and **D**)

**Fig. 5:**
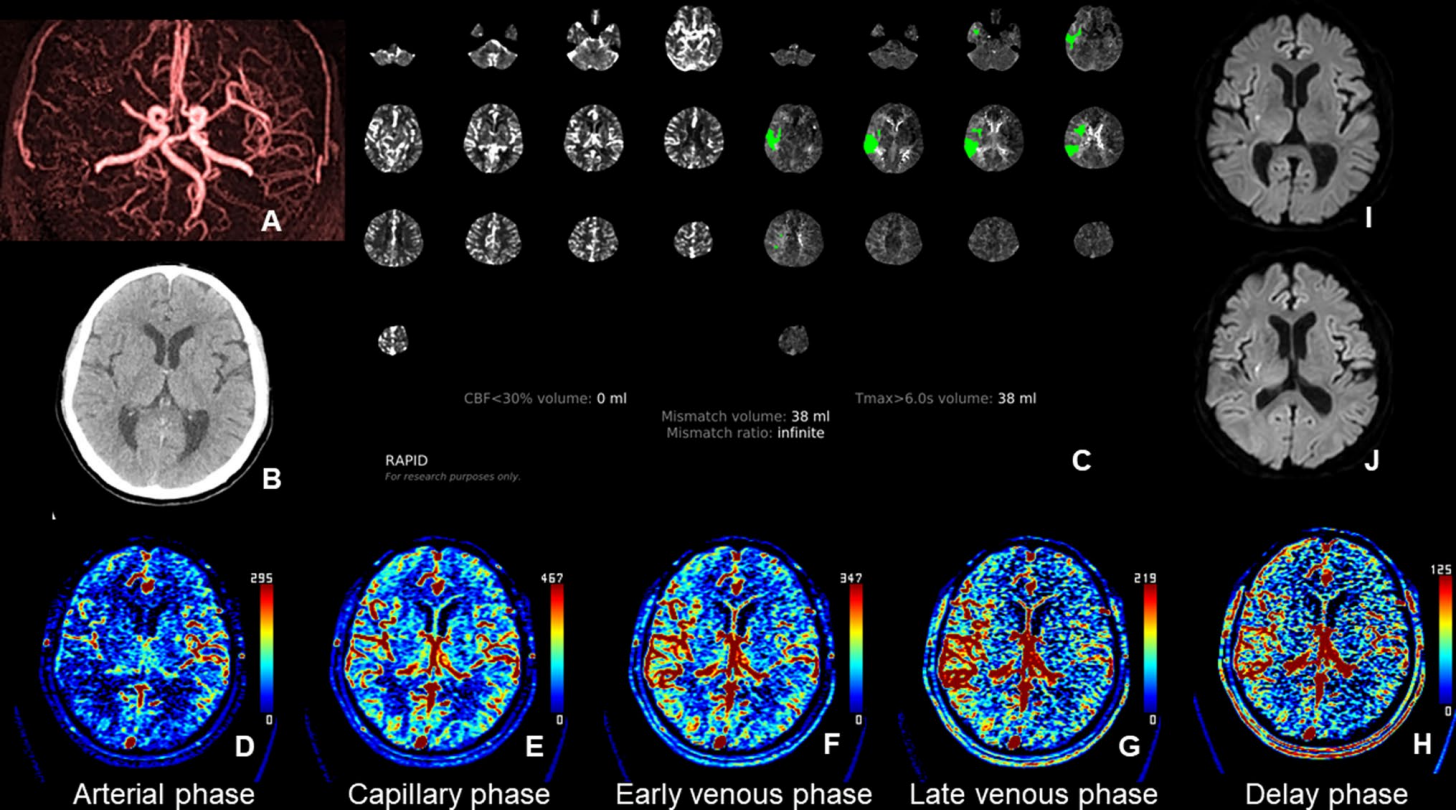
Images of a 76-year-old man with occlusion of the right middle cerebral artery (MCA) demonstrated on CT angiography (**A**) derived from CT perfusion (CTP). The patient had a premorbid modified Rankin Scale (mRS) score of 0 and an National Institutes of Health Stroke Scale (NIHSS) score of 3 at admission. He was managed with conservative treatment, and his 90-day mRS score was 2. The brain CT scan at admission (**B**) showed no abnormal findings. CT perfusion analysis using RAPID software (RAPID, RapidAI®, Menlo Park, CA, USA) (**C**) revealed a large penumbra, indicating potential for infarct growth. However, the CT collateral map (**D**–**H**) derived from CTP at admission displayed excellent collateral perfusion (collateral perfusion score of 5: no or minimal collateral perfusion delay in the ischemic MCA territory during the capillary phase), suggesting minimal risk of infarct progression. A small acute infarct signal was observed in the right striatocapsular region on the admission DWI (**I**), and the baseline lesion showed no significant growth on DWI performed on Day 1 (**J**)

**Fig. 6:**
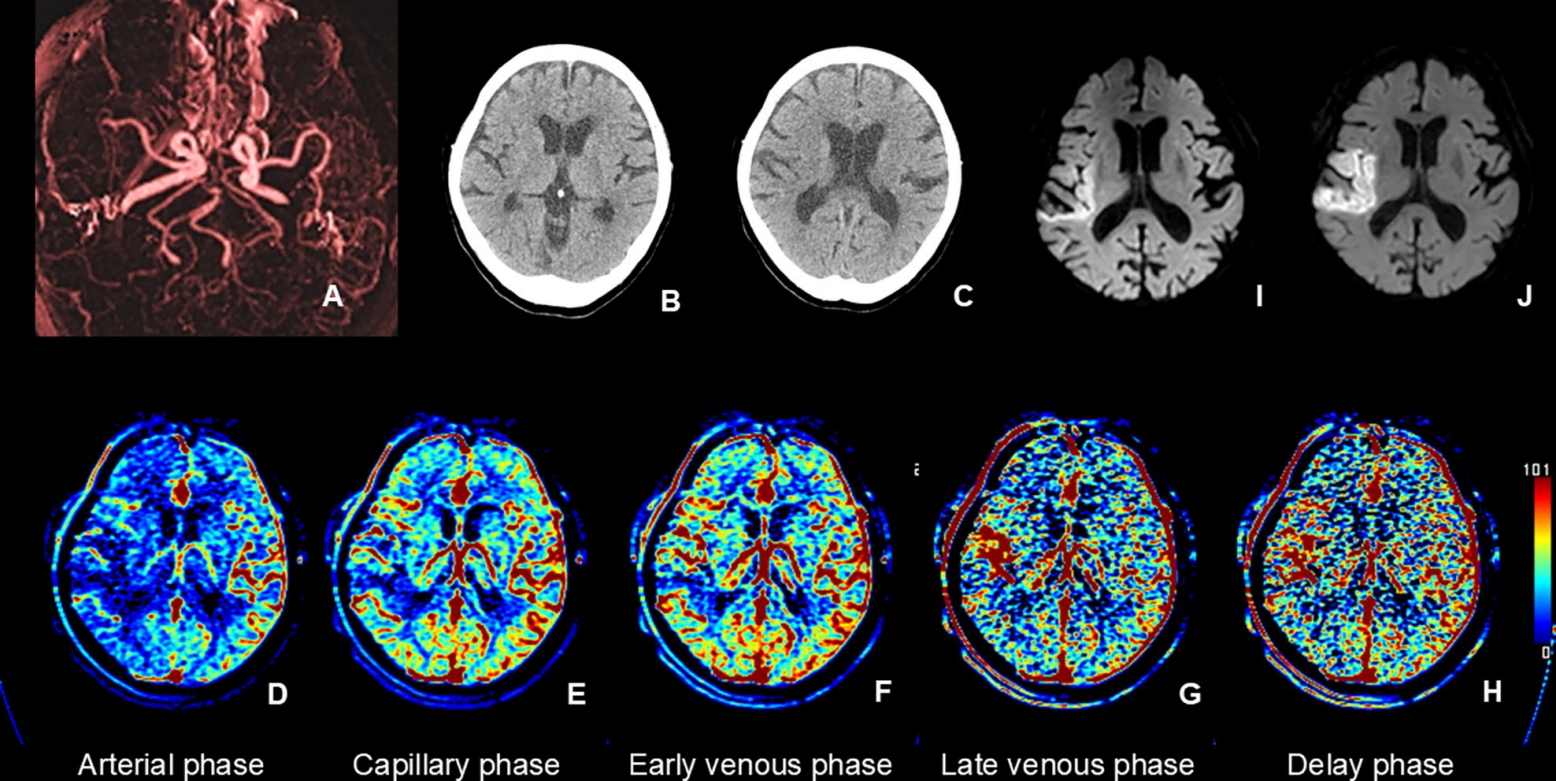
Images of an 87-year-old woman with occlusion of the right middle cerebral artery (MCA) demonstrated on CT angiography (**A**) derived from CT perfusion. The patient had a premorbid modified Rankin scale score of 0 and presented with the National Institutes of Health Stroke Scale score of 9 upon admission. Conservative treatment was administered. At 90 days, the patient's mRS score was 3. Initial brain CT images (**B** and **C**) showed ambiguous differentiation between the cortical gray matter and white matter in the right insula, making it difficult to accurately determine the extent of the baseline infarct. The CT collateral map (**D**–**H**), generated from the admission CT perfusion, indicated a moderate collateral perfusion status (collateral perfusion score of 3: collateral perfusion delay affecting less than half of the middle cerebral artery territory during the capillary and early venous phases), suggesting potential infarct growth. Diffusion-weighted imaging (DWI) (I), performed immediately after the CT scan, showed acute infarct signals within the right MCA territory. The extent of the DWI lesion at admission (**I**) corresponded closely with the hypoperfused area observed in the early venous phase (**F**) of the CT collateral map. On Day 1, follow-up DWI (**J**) revealed that the baseline lesion had expanded to a size similar to the hypoperfused area seen in the capillary phase (**E**) of the CT collateral map at admission. These findings suggest that even in the absence of DWI, the CT collateral map could potentially be used to identify the baseline lesion and predict infarct growth, as well as estimate the potential expansion of the infarcted area

The interrater correlation among the three raters demonstrated excellent agreement in estimating the CMC and CMEV lesion volumes (CCC = 0.92; 95% CI: 0.87, 0.99 and CCC = 0.97; 95% CI: 0.95, 0.99, respectively). In 110 patients, the median bDWI lesion volume was 12.6 mL (IQR, 2.9–34.8 mL), the median CMEV lesion volume was 14.2 mL (IQR, 0.1–32.3 mL), and the median CBF < 30% lesion volume was 7.5 mL (IQR, 0.1–27.0 mL). The agreement between bDWI lesion volumes and CMEV lesion volumes (CCC = 0.97; 95% CI: 0.95, 0.98) was greater than the agreement between bDWI lesion volumes and CBF < 30% lesion volumes (CCC = 0.76; 95% CI: 0.60, 0.91) (Fig. 3 and 4). The difference in the CCC between CBF < 30% lesion volume and CMEV lesion volumes was −0.22 (95% CI: − 0.38—−0.07). This difference was statistically significant, as the 95% CIs of the difference in CCCs did not include 0 (Table 3). Linear regression analysis also indicated greater predictive value of CMEV lesion volumes than CBF < 30% lesion volumes for bDWI lesion volumes (Table 3). Fifteen patients (mean age: 70 years ± 15 years, 53.3% women) were included in the subgroup analysis. An excellent CP grade was obtained in 6 patients (40.0%), a good grade in 2 patients (13.3%), an intermediate to good grade in 5 patients (13.3%), an intermediate to poor grade in 2 patients (13.3%), and no patients had poor or very poor grades (0.0%). The median lesion growth ratio, mismatch ratio, and collateral ratio in the 8 patients with excellent and good CP grades were 1.0 mL (IQR, 1–1.1), 1.0 mL (IQR, 1–32.5), and 1.0 (IQR, 1–1), respectively. The bDWI lesions of these patients did not exhibit lesion growth. However, the mismatch ratio predicted lesion growth in 3 of these patients (Fig. 5). In 7 patients with intermediate to good and intermediate to poor CP grades, the median bDWI and f/u DWI lesion volumes were 21.7 mL (IQR, 14.3–43.6 mL) and 63.8 mL (IQR, 36.3–74.3 mL), respectively, the median CMEV and CMC lesion volumes were 19.6 mL (IQR, 16.6–38.1 mL) and 56.3 mL (IQR, 34.2–70.1 mL) respectively, and the median CBF < 30% and Tmax > 6 s lesion volumes were 25.0 mL (IQR: 14.5–30.5 mL) and 81.0 mL (IQR: 54.0–102.0 mL), respectively. The median lesion growth ratio, mismatch ratio, and collateral ratio were 2.6 mL (IQR, 1.7–3.5), 3.2 mL (IQR, 3.0–4.7), and 2.6 mL (IQR, 1.6–3.2), respectively. bDWI lesions of these patients showed lesion growth. The agreement between the collateral ratio and lesion growth ratio (CCC = 0.86; 95% CI: − 0.05, 0.03) was significantly greater than the agreement between the mismatch ratio and lesion growth ratio (CCC = 0.12; 95% CI: − 0.04, 0.50) (Fig. 6). The agreement between f/uDWI lesion volumes and CMC lesion volumes (CCC = 0.89; 95% CI: 0.77, 0.95) was greater than the agreement between f/uDWI lesion volumes and Tmax > 6 s lesion volumes (CCC = 0.65; 95% CI: 0.38, 0.87) (Fig. 4). The difference in the CCCs was not significant; however, linear regression analysis showed a significantly greater predictive value of CMC lesion volumes than of Tmax > 6 s lesion volumes for f/uDWI lesion volumes (Table 3) (Fig. 6).

**Table 3:** Performance of the perfusion values (CBF < 30%^*^ and *T*max > 6 s^†^) and the collateral map values (hypoperfusion lesion volumes in the early venous and capillary phases) for the prediction of baseline and follow-up diffusion-weighted imaging (DWI) lesion volumes

	For the prediction of baseline DWI lesion volume^‡^		For the prediction of follow-up DWI lesion volume^§^	
	Lesion volume with CBF < 30%	Hypoperfused lesion volume in the CMEV	Lesion volume^|^ with Tmax > 6 s	Hypoperfused lesion volume in the CMC
CCC (95% CI)	0.76 (0.60, 0.91)	0.97 (0.95, 0.98)	0.65 (0.38, 0.87)	0.89 (0.77, 0.95)
Difference in the CCCs	− 0.22 (− 0.38, − 0.07)		− 0.24 (− 0.53, 0.06)	
Beta (standard error)	− 0.002 (0.025)	1.102 (0.034)	− 0.077 (0.236)	1.516 (0.252)
*P* value of Beta	0.929	< 0.001	0.758	0.002

Beta, beta coefficient in linear regression analysis; CCC, concordance correlation coefficient; CI, confidence interval; CMC, capillary phase of the CT collateral map; CMEV, early venous phase of the CT collateral map

^*^Perfusion threshold of < 30% of the contralateral mean cerebral blood flow rate

^†^Perfusion threshold of > 6 s of the contralateral mean time-to-maximum

^‡^Analysis of a total of 110 patients included in this study. The mean overall patient age was 71 years ± 14 (standard deviation) years (50 men and 60 women)

^§^Analysis of subgroups (7 of 110 patients) who met the following inclusion criteria: (1) patients with follow-up DWI and angiography within 7 days, (2) patients who presented with unchanged stenoocclusive arterial lesions on follow-up angiography, and (3) patients with intermediate to good, intermediate to poor, poor, or very poor collateral perfusion status

## Discussion

Our study demonstrated the reliability of CP estimation using the CT collateral map, as evidenced by its excellent correlation with CP estimation using the MRA collateral map (weighted κ = 0.93; 95% CI: 0.90, 0.97) in patients with acute anterior circulation ischemic stroke. The CMEV lesion volume at baseline lesion assessment was superior to that of CBF < 30% lesion volume (*P* < 0.001). While caution is warranted due to the limited sample size, the CMC lesion volume proved superior to Tmax > 6 s lesion volume for predicting final infarct lesions (*P* = 0.002) and demonstrated that the collateral ratio predicts the lesion growth ratio more accurately than the mismatch ratio (CCC = 0.86; 95% CI: − 0.05, 0.03 for collateral ratio and CCC = 0.12; 95% CI: − 0.04, 0.50 for mismatch ratio).

The primary objective of recanalization therapies is to preserve viable brain tissue to enhance functional outcomes. Collateral circulation is the key source of blood flow that sustains the ischemic brain beyond an arterial blockage. This circulation varies significantly between individuals and is a critical factor in determining the extent of the final infarct and the overall clinical outcomes. Roh et al. created the MRA collateral map, which visualizes CP status in an individual patient’s hemodynamic phases [10]. This map allows for an intuitive assessment of the timing and volume of blood flow reaching the ischemic brain in each patient. Kim et al. identified a direct correlation between the CP grade on the MRA collateral map and the functional outcomes of patients with AIS in the anterior circulation [11]. Recently, Yi et al. demonstrated that the MRA collateral map could be used to make personalized predictions about lesion growth and the extent of salvageable brain tissue by evaluating baseline and final lesions through CMEV and CMC lesion volumes [12]. Lu commented that these findings of the MRA collateral map may enable the patient-specific application of recanalization therapies and improve the results of the treatments [23].

CT is the first-line tool for evaluating patients with AIS, offering significant advantages due to its easy and widespread accessibility. Nevertheless, it has a critical drawback in its inaccuracy in assessing baseline lesions, which also poses a major obstacle to penumbra estimation [24, 25]. This can result in eligible patients for recanalization therapies being excluded from treatments, and patients with large infarctions may undergo treatments, leading to futile interventions. In the wake of the DAWN and DEFUSE3 trials [21, 22], CBF < 30% and Tmax > 6 s have emerged as pivotal values for estimating the infarct core and penumbra, respectively. However, the reliability of the infarct core and penumbra remains constrained compared to DWI, and CBF < 30% and Tmax > 6 s values can fluctuate depending on the postprocessing algorithm used [16, 17]. The primary issue arises because perfusion imaging provides a calculated snapshot based on single numeric thresholds, which fails to capture the complex spatial and temporal nature of ischemia in patients with varying hemodynamic conditions [26].

The CT collateral map is generated using dynamic contrast information according to each patient’s hemodynamics, similar to the MRA collateral map. Our study demonstrated that the CT collateral map derived from CT perfusion serves a similar function to the MRA collateral map derived from DCE-MRA in CP estimation and assessment of baseline and final lesions, performing better than a CBF < 30% and a Tmax > 6 s. We briefly introduce simultaneous arteriography, venography, and dynamic angiography derived from CT perfusion, which, when combined with a CT collateral map, is a CT protocol composed of noncontrast brain CT and CT perfusion that provides accurate information on the baseline and final lesions, causative vessel, and collateral perfusion state in AIS patients.

The current guidelines for recanalization therapies may exclude patients with mild symptoms (NIHSS < 6) who are at risk of disease progression due to lesion growth, as well as patients with large baseline lesions (ASPECTS < 6 or ischemic core volume > 70 ~ 100 mL) who could benefit from recanalization [27]. It has been reported that futile recanalization is observed in nearly half of patients after endovascular thrombectomy [28]. Previous studies demonstrated that a very poor CP grade on the MRA collateral map was an independent predictor of futile recanalization and symptomatic hemorrhagic transformation [8, 9]. The demographic findings of this study (Table 2) revealed that most patients with CP scores of 5 and 4 exhibited mild symptoms, and based on the results of this study, it is expected that their baseline lesion will not grow. However, for patients with mild symptoms and a CP score of 3, baseline lesions may increase, making them eligible for recanalization therapies. Additionally, patients with bDWI lesion volumes over 100 mL were classified with a CP score 0 (very poor collateral perfusion score), demonstrating that collateral perfusion estimation can help identify large stroke patients who are likely to experience futile recanalization. These assumptions need to be validated through further studies. However, considering that baseline lesion volume and symptom severity are consequences of ischemia, an accurate assessment of collateral perfusion, the underlying cause, is expected to facilitate the precise application of recanalization therapies, improving treatment outcomes and reducing the incidence of futile treatments. This study suggests the potential of the CT collateral map in contributing to this effort.

There were limitations to our study. A retrospective study can be a limitation. However, in this study, all patients underwent a CT collateral map, CT perfusion, and MRA collateral map, and the performance of each imaging modality was compared. Therefore, the impact of selection bias inherent to a retrospective study on the study results is likely minimal. We manually segmented the CMC and CMEV lesions, which could be more delicate, while the software automatically segmented those with a CBF < 30% and a T max > 6 s. This may have led to an overestimation of the differences between collateral maps and conventional perfusion thresholds. If automatic analysis technology for the CT collateral map is developed, it is expected to enhance the utility of the CT collateral map and enable direct comparison with the automatic analysis results of other modalities. The number of patients included in the final lesion assessment was too small. It is very difficult to include patients who meet the eligibility criteria, especially in the early time window, when most patients are eligible for recanalization therapies and successful recanalization is achieved in most. The final lesion assessment was not the main focus of this study due to the small sample size; it was conducted to understand the trends in previous studies on MRA collateral maps, which are important factors in the evaluation of patients with AIS.

In conclusion, the clinical feasibility of the CT collateral map is demonstrated by its almost perfect agreement with the MRA collateral map in CP grading and baseline lesion assessment among patients with acute anterior circulation ischemic stroke. The CT collateral map may improve the accuracy of CT-based work-up in AIS patients and facilitate individualized application of recanalization therapies.

## Abbreviations

AIS: Acute ischemic stroke
bDWI: Baseline diffusion-weighted imaging
CBF: Cerebral blood flow rate
CCC: Concordance correlation coefficient
*CI*: Confidence interval
CMC: Capillary phase of the CT collateral map
CMEV: Early venous phase of the CT collateral map
*CP*: Collateral perfusion
DASAN: Database of acute ischemic stroke analysis network
DCE-MRA: Dynamic contrast-enhanced magnetic resonance angiography
DWI: Diffusion-weighted imaging
f/uDWI: Follow-up diffusion-weighted imaging
IQR: Interquartile range
κ: Kappa
NIHSS: National Institutes of Health Stroke Scale
Tmax: Time-to-maximum
s: Second
